# The association between triglyceride-glucose index and related parameters and risk of tuberculosis infection in American adults under different glucose metabolic states: a cross-sectional study

**DOI:** 10.1186/s12889-025-21793-6

**Published:** 2025-03-11

**Authors:** Min Qi, Runjuan Qiao, Jian-Qing He

**Affiliations:** 1 Department of Pulmonary and Critical Care Medicine, West China Hospital, Sichuan University, No. 37 Guoxue Alley, Chengdu, 610041 People’s Republic of China; 2 State Key Laboratory of Respiratory Health and Multimorbidity, West China Hospital, Sichuan University, Chengdu, 610041 People’s Republic of China; 3 General Practice Ward/International Medical Center Ward, General Practice Medical Center, West China Hospital, Sichuan University, Chengdu, 610041 People’s Republic of China

**Keywords:** Triglyceride-glucose index, Insulin resistance, Latent tuberculosis infection, NHANES

## Abstract

**Background:**

Tuberculosis (TB) and diabetes mellitus (DM) are known to influence each other, with insulin resistance playing a pivotal role. The relationship between the triglyceride-glucose (TyG) index and its derived indices with the incidence of TB infection across varying glucose metabolic statuses is not well defined.

**Methods:**

This cross-sectional study utilized data from the 2011–2012 National Health and Nutrition Examination Survey. Weighted multivariable regression analysis was employed to explore the correlation between TyG and associated parameters with the incidence of TB infection within different categories of glucose metabolism. Interaction analyses and restricted cubic splines were utilized to assess potential heterogeneity in these associations and to explore the link between TyG and its derivatives with the occurrence of TB infection.

**Results:**

The study included 4823 participants, of which 668 had TB infection. In individuals with normal glucose tolerance (NGT), the TyG index (OR 2.17, 95%CI 1.40–3.35), TyG-WC (OR 1.01, 95%CI 1.00-1.01), and TyG-BMI (OR 1.02, 95%CI 1.00-1.04) were correlated with TB infection (all *P* < 0.05). Among participants with impaired fasting glucose (IFG), TyG (OR 57.10, 95%CI 1.17-278.66), TyG-WC (OR 1.02, 95%CI 1.00-1.05), TyG-WHtR (OR 872.94, 95%CI 43.31-17592.72) were significant associated with TB infection (all *P* < 0.05). However, in those with impaired glucose tolerance (IGT) and DM, TyG and its related parameters did not show an association with TB infection (*P* > 0.05). The sensitive analysis, converting the TyG index from a continuous variable to a categorical variable (quartiles), revealed an association between the TyG index and an increase risk of TB infection in the NGT and IGT group (quartile 4: OR 2.45 (1.31–4.60) and 761.33 (10.54–54999.02), respectively). No significant association between the TyG index and TB infection was observed in DM and IFG groups.

**Conclusions:**

In participants with NGT and IFG, the levels of the TyG index and its associated parameters were correlated with TB infection. A higher TyG index was independently linked to an increased likelihood of TB infection in individuals with NGT and IGT, but not in DM and IFG.

## Background

Tuberculosis (TB), an infectious disease caused by Mycobacterium tuberculosis, is a primary contributor to global mortality and represents a substantial disease burden globally [1]. Latent tuberculosis infection (LTBI), serves as a reservoir for the disease, continually fueling the emergence of new cases. Certain populations are at an elevated risk of transitioning from LTBI to active TB, with an estimated one-quarter of the global population being in a state of LTBI [2]. It is reported that 5–10% of individuals with LTBI will eventually develop active TB during their lifetime [3]. The unchecked burden of LTBI threatens the realization of global targets aimed at TB elimination. Consequently, the programmatic identification and management of LTBI are essential components in the strategy to control and ultimately eradicate TB [4].

Individuals with diabetes mellitus(DM) are more frequently affected by infectious diseases, which potentially increases morbidity and mortality [5]. The presence of DM is known to elevate the risk of developing TB by approximately threefold, and it also significantly amplifies the risk of mortality during TB treatment, as well as the likelihood of other adverse outcomes [6]. Moreover, a meta-analysis has indicated a correlation between DM and an enhanced susceptibility to LTBI [7, 8]. Additionally, a study enrolled 890 microbiologically confirmed TB patients reported that both pre-DM (sometimes termed non-diabetes dysglycemia or intermediate hyperglycemia) [6] and DM are linked to a higher bacterial load [9]. However, the association between dysglycemia and TB infection has not been fully elucidated.

Insulin resistance (IR) is a state characterized by the body’s diminished sensitivity to insulin, resulting in an impaired capacity for glucose uptake by peripheral tissues and a dampened inhibitory effect on hepatic glucose production, which ultimately culminates in hyperglycemia [10]. The triglyceride-glucose (TyG) index has emerged as a robust indicator for the assessment of IR, offering a potentially more reliable evaluation compared to traditional tools such as the homeostasis model assessment of IR (HOMA-IR) [11, 12]. TyG index, along with its derived parameters—including TyG–waist circumference (TyG-WC), TyG–body mass index (TyG-BMI), and TyG–waist-to-height ratio (TyG-WHtR)—has gained recognition as alternative indicators of IR in the context of various metabolic disorders [13, 14], these indices have demonstrated predictive value in conditions such as nonalcoholic fatty liver disease [14] and cardiovascular disease [15]. However, the relationship between the TyG index and its related parameters with the risk of TB infection, particularly across varying states of glucose metabolism, remains to be fully understood. The aim of our study is to explore the potential prognostic significance of the TyG index and its associated parameters in predicting the likelihood of TB infection in the adult U.S. population, considering diverse glucose metabolic statuses.

## Materials and methods

### Study design and population

The National Health and Nutrition Examination Survey (NHANES) is designed to provide a nationally representative snapshot of the health and nutritional status of the non-institutionalized civilian population in the United States, NHANES utilizes a sophisticated, stratified, multistage probability cluster sampling methodology. This dynamic survey is conducted biennially, offering a series of independent cross-sectional evaluations. NHANES encompasses standardized in-home interviews, detailed health examinations at dedicated mobile examination centers, and a suite of laboratory tests. These components are harmoniously integrated to assess a broad spectrum of medical and physiological parameters among participants.

**Fig. 1:**
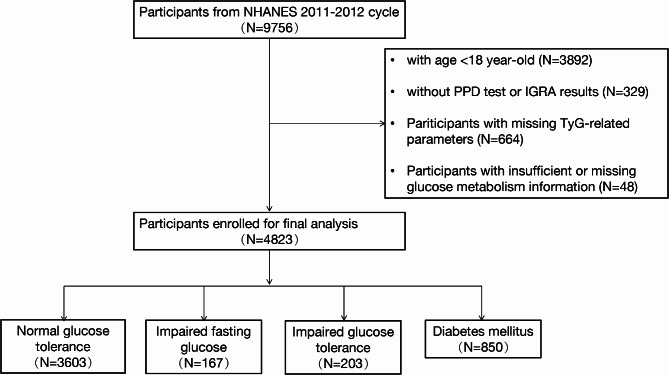
Flowchart of the sample selection from National Health and Nutrition Examination Survey (NHANES) 2011–2012

In this study, we extracted data from NHANES spanning the years 2011to 2012, including a total of 9756 anonymous records. The following exclusion criteria were applied: [1] individuals under the age of 18 [2], those without Purified Protein Derivative (PPD) test or Interferon-Gamma Release Assays (IGRA) results [3], participants with incomplete data regarding TyG-related parameters, and [4] individuals with inadequate or absent information on glucose metabolism. Ultimately, a total of 4823 participants were included for analysis (Fig. 1). The NHANES study has gained ethical approval by the Institutional Review Board at the National Center for Health Statistics and written informed consent was obtained from all participants.

### Laboratory tests and clinical data

In our study, we accessed a comprehensive array of clinical information from the NHANES database, including age, gender, education level, race, poverty income ratio (PIR), smoking status, and alcohol consumption, creatinine, total cholesterol, triglycerides, HbA1c, albumin, glucose were obtained from the original database. In addition, measurements such as height (cm), waist circumference (cm), and BMI were recorded. Disease status, including diabetes, hypertension, and chronic kidney disease (CKD), was also documented. Fasting venous blood samples were collected and measured in the laboratory. The NHANES study protocol specifies that laboratory assessments requiring blood samples are conducted exclusively with participants aged 12 years and older. Eligible subjects must have fasted for a period of at least 8.5 h up to, but not exceeding, 24 h prior to the morning blood draw.

### Assessment of TyG-related index

We selected the TyG index as the primary exposure variables, calculated using the formulation: Ln [triglycerides (mg/dl) × fasting glucose (mg/dl)/2]. Triglycerides concentration was measured via the Roche Modular *P* and Roche Cobas 6000 analyzers. Fasting plasma glucose levels were determined using the Roche/Hitachi Cobas C 501 analyzer based on the hexokinase-mediated reaction.

And TyG related parameters were calculated as follows: TyG-WC = TyG×waist circumference (cm), TyG-BMI = TyG×BMI (kg/m^2^), WHtR = waist circumference (cm)/height (cm), TyG-WHtR = TyG×WHtR.

### Definition of glucose metabolism status

DM was defined by self-reported doctor-diagnosed diabetes, medication or insulin use for diabetes, or glycated hemoglobin (HbA1c) ≥ 6.5%, fasting blood glucose ≥ 7.0 mmol/L or 2 h oral glucose tolerance test (OGTT) glucose levels ≥ 11.1 mmol/L.

Impaired fasting glucose (IFG) was identified in participants whose fasting blood glucose levels were between 5.6 and 6.9 mmol/L but not meet the diagnostic criteria for diabetes and 2 h OGTT glucose levels < 7.8 mmol/L.

Impaired glucose tolerance (IGT) was defined by fasting blood glucose levels < 5.6 mmol/L and 2 h OGTT glucose levels between 7.8 and 11.0 mmol/L, without fulfilling the criteria for a diabetes diagnosis.

Normal glucose tolerance (NGT) was defined by fasting blood glucose levels < 5.6 mmol/L and 2 h OGTT glucose levels < 7.8 mmol/L.

### Assessment of LTBI

For the assessment of LTBI, participants in the NHANES study underwent a skin test using a commercially available antigen, PPD, specifically tubersol. A precise volume of 0.1 ml (equivalent to 5 international units) of the designated PPD was administered by trained NHANES technicians. These technicians, blinded to the participant’s medical history and potential TB contact, measured the skin reaction 46–76 h post-administration. Additionally, NHANES participants underwent an IGRA test, the QuantiFERON-TB Gold In-Tube (QFT-GIT), an FDA-approved method for detecting TB infection. The QFT-GIT utilized specialized blood collection tubes, including a Nil control tube (negative control), a TB Antigen tube, and a Mitogen tube (positive control), for the collection of whole blood via venipuncture. After collection, the tubes were thoroughly mixed to ensure complete antigen-antibody interaction and then incubated at a controlled temperature of 37 °C ± 1 °C for 16–24 h. Following incubation, plasma was extracted, and an enzyme-linked immunosorbent assay was employed to quantify the interferon-gamma (IFN-γ) levels in response to the peptide antigens. A positive TST was defined by an induration size of ≥ 5 mm, a criterion commonly applied for the adults US population, excluding those with special risk factors. For a positive interpretation of the QFT-GIT, we adhered to the NHANES guidelines, which included the following criteria: (1) The Nil value ≤ 8.0 IU of gamma interferon (IF)/ml, (2) The TB antigen value minus the Nil value ≥ 0.35 IU IF/ml, (3) The TB antigen value minus the Nil value ≥ 25% of the Nil value. An inadequate response to mitogen (< 0.5 IU/mL), alongside a negative response to TB antigens, was considered indeterminate. Individuals with positive TST or QFT-GIT results were considered as having LTBI.

### Statistical analyses

The statistical analysis was conducted utilizing R software (version 4.3.2), and data extraction and merging were performed with nhanesR package (version 0.9.5.0). Continuous variables were presented as weighted means with standard deviation, whereas categorical variables were expressed as estimated proportions. One-way ANOVA test was applied to assess intergroup differences in continuous variables, and chi-square tests was employed to evaluate differences in categorical variables.

To investigate the association between TyG and related parameters with LTBI, multivariable logistic regression models were utilized. Additionally, we employed restricted cubic splines (RCS) to explore the nonlinear relationship between TyG and its derivatives with LTBI. In sensitivity analyses, the TyG index was categorized into quartiles to assess the robustness of the results, and the likelihood of across these quartiles was evaluated. Statistical difference was determined using a two-side *P* value threshold of less than 0.05.

## Results

### Baseline characteristics of participants in different glucose metabolic groups

This study included 4823 participants, representing 200 million U.S. non-institutionalized residents. As shown in Table 1, those with NGT or IGT were younger, and with lower levels of HbA1c, BMI, fasting blood glucose, creatinine, triglycerides, TyG index, TyG-WC, TyG-BMI, TyG-WHtR, CKD, hypertension rates, and with higher albumin, cholesterol levels and alcohol consumption rate (all *P* value < 0.05). As for the LTBI rate, participants with IGT had the highest, and NGT participants had the lowest. Gender, smoking status, and PIR exhibited no significant differences across glucose metabolism categories.

**Table 1:** Weighted baseline characteristics in different glucose metabolic groups

Variable	All participants	DM	IFG	IGT	NGT	*P* value
Age (year), mean (SD)	46.14(0.82)	58.47(0.80)	51.59(1.45)	53.88(1.19)	43.46(0.90)	< 0.0001
HbA1c, mean (SD)	5.63(0.02)	7.22(0.07)	5.65(0.03)	5.51(0.04)	5.38(0.01)	< 0.0001
BMI (kg/m2), mean (SD)	28.58(0.23)	32.97(0.34)	31.18(0.44)	29.12(0.54)	27.71(0.20)	< 0.0001
Glucose(mg/dl), mean (SD)	98.56(0.88)	148.30(3.84)	108.31(0.46)	96.16(1.85)	90.18(0.26)	< 0.0001
Albumin(g/dl), mean (SD)	4.33(0.01)	4.19(0.01)	4.27(0.03)	4.25(0.05)	4.36(0.01)	< 0.0001
Creatinine(mg/dl), mean (SD)	0.88(0.01)	0.98(0.04)	0.92(0.02)	0.87(0.02)	0.87(0.01)	0.04
Triglycerides(mg/dl), mean (SD)	1.71(0.05)	2.42(0.13)	1.83(0.17)	1.70(0.15)	1.59(0.05)	< 0.001
Cholesterol(mg/dl), mean (SD)	191.96(1.06)	186.25(2.29)	196.23(5.78)	200.15(5.34)	192.22(0.99)	0.02
TyG, mean (SD)	4.19(0.03)	4.87(0.06)	4.44(0.08)	4.20(0.06)	4.07(0.03)	< 0.0001
TyG-WC, mean (SD)	416.67(5.58)	542.51(10.89)	475.50(12.73)	430.70(9.98)	392.68(4.90)	< 0.0001
TyG-BMI, mean (SD)	121.18(1.73)	161.39(3.52)	139.47(3.71)	123.14(3.20)	113.68(1.42)	< 0.0001
TyG-WHtR, mean (SD)	2.47(0.03)	3.25(0.06)	2.79(0.07)	2.54(0.05)	2.32(0.03)	< 0.0001
Sex, *n* (%)						0.07
Female	2378(50.22)	407(48.66)	65(37.31)	98(47.79)	1808(51.24)	
Male	2445(49.78)	443(51.34)	102(62.69)	105(52.21)	1795(48.76)	
Races, *n* (%)						< 0.001
Non-Hispanic Black	1239(10.84)	271(15.66)	44(9.72)	34(6.48)	890(10.36)	
Mexican American	504(8.05)	100(10.00)	15(5.08)	24(8.01)	365(7.88)	
Non-Hispanic white	1772(67.03)	252(57.21)	73(76.38)	83(72.74)	1364(67.84)	
Other	1308(14.08)	227(17.13)	35(8.83)	62(12.77)	984(13.92)	
Educational levels, *n* (%)						< 0.001
College graduate or above	1184(30.72)	149(19.45)	38(28.87)	52(33.32)	945(32.48)	
High school graduate or less	2244(38.00)	489(53.15)	85(38.03)	97(36.39)	1573(35.63)	
Some college or AA degree	1394(31.28)	211(27.40)	44(33.09)	54(30.29)	1085(31.88)	
Alcohol user, *n* (%)						0.02
No	649(9.48)	146(14.82)	21(7.44)	30(9.78)	452(9.62)	
Yes	3762(83.31)	641(85.18)	137(92.56)	156(90.22)	2828(90.38)	
Smoke, *n* (%)						0.06
Former	1040(23.78)	266(32.27)	40(27.29)	58(29.53)	676(22.92)	
Never	2598(53.58)	429(49.60)	85(49.45)	106(53.64)	1978(56.85)	
Now	923(19.24)	146(18.13)	42(23.26)	35(16.84)	700(20.22)	
CKD, *n* (%)						< 0.0001
No	4006(86.36)	512(64.29)	135(85.41)	178(91.14)	3181(89.93)	
Yes	801(13.43)	334(35.71)	31(14.59)	25(8.86)	411(10.07)	
Hypertension, *n* (%)						< 0.0001
No	2933(64.37)	258(30.90)	71(45.34)	105(57.74)	2499(71.07)	
Yes	1890(35.63)	592(69.10)	96(54.66)	98(42.26)	1104(28.93)	
PIR, *n* (%)						0.11
<1	1128(16.29)	223(21.43)	34(12.15)	43(14.20)	828(17.12)	
>4	1106(33.05)	131(25.22)	47(40.61)	44(33.03)	884(36.68)	
1–4	2201(44.54)	419(53.36)	72(47.25)	96(52.77)	1614(46.21)	
LTBI, *n* (%)						0.01
No	4155(92.41)	700(89.02)	143(91.15)	165(87.49)	3147(93.30)	
Yes	668(7.59)	150(10.98)	24(8.85)	38(12.51)	456(6.70)	

DM, diabetes mellitus; IFG, impaired fasting glucose; IGT, impaired glucose tolerance; NGT, normal glucose tolerance; PIR, poverty income ratio; CKD, chronic kidney disease; BMI, body mass index; TyG, triglyceride-glucose index; LTBI, latent tuberculosis infection

### Baseline characteristics of participants in different TB infection status

In our study, a total of 668 participants were identified with TB infection (Table 2). We compared the characteristics of participants with LTBI and those without LTBI. Participants with TB infection exhibited higher levels of HbA1c, fasting glucose, as well as elevated TyG index value. Additionally, a higher proportion of males and individuals with dysregulated glucose metabolism were observed in the TB infection group. This group also displayed lower rates of alcohol consumption compared with their non-LTBI counterparts. However, no significant differences were noted in terms of age, BMI, TyG related parameters, smoke status, CKD and hypertension between the LTBI and non-LTBI groups.

**Table 2:** Weighted baseline characteristics in TB infection and non-infection group

Variable, mean (SD)	All participants (*n* = 4823)	Non-LTBI (*n* = 4155)	LTBI (*n* = 668)	*P* value
Age (year), mean (SD)	45.97(0.81)	45.98(0.85)	45.85(1.30)	0.92
HbA1c, mean (SD)	5.63(0.02)	5.61(0.03)	5.83(0.04)	< 0.001
BMI (kg/m2), mean (SD)	28.60(0.23)	28.62(0.24)	28.32(0.36)	0.45
Glucose(mg/dl), mean (SD)	98.40(0.85)	98.03(0.90)	102.96(1.31)	0.004
Albumin(g/dl), mean (SD)	4.32(0.01)	4.32(0.01)	4.32(0.02)	0.98
Creatinine(mg/dl), mean (SD)	0.88(0.01)	0.88(0.01)	0.85(0.02)	0.08
Triglycerides(mg/dl), mean (SD)	1.71(0.04)	1.71(0.05)	1.81(0.08)	0.21
Cholesterol(mg/dl), mean (SD)	192.34(1.06)	192.31(1.11)	192.68(2.17)	0.87
TyG, mean (SD)	4.19(0.03)	4.18(0.03)	4.29(0.03)	0.01
TyG_WC, mean (SD)	416.85(5.47)	416.58(5.79)	420.16(6.06)	0.62
TyG_BMI, mean (SD)	121.25(1.70)	121.13(1.80)	122.69(2.26)	0.55
TyG_WHtR, mean (SD)	2.47(0.03)	2.47(0.04)	2.54(0.04)	0.1
Sex, *n* (%)				0.01
Female	2426(50.75)	2136(51.31)	290(43.86)	
Male	2445(49.25)	2065(48.69)	380(56.14)	
Races, *n* (%)				< 0.0001
Non-Hispanic Black	1254(10.90)	1083(10.41)	171(16.88)	
Mexican American	510(8.08)	402(6.91)	108(22.47)	
Non-Hispanic white	1320(14.09)	974(11.78)	346(42.49)	
Other	1787(66.94)	1742(70.91)	45(18.16)	
Educational levels, *n* (%)				< 0.0001
College graduate or above	1198(30.80)	1050(31.64)	148(20.52)	
High school graduate or less	2256(37.82)	1873(36.17)	383(58.10)	
Some college or AA degree	1416(31.38)	1278(32.19)	138(21.39)	
Alcohol user, *n* (%)				0.002
No	658(9.54)	541(9.86)	117(15.74)	
Yes	3793(83.15)	3305(90.14)	488(84.26)	
Smoke, *n* (%)				0.96
Former	1047(23.67)	896(24.52)	151(24.15)	
Never	2636(53.88)	2258(55.77)	378(55.58)	
Now	926(19.08)	807(19.70)	119(20.26)	
Glucose metabolism status, *n* (%)				0.01
DM	850(12.64)	700(12.31)	150(18.50)	
IFG	167(3.79)	143(3.78)	24(4.47)	
IGT	203(4.34)	165(4.16)	38(7.23)	
NGT	3603(78.16)	3147(79.75)	456(69.80)	
CKD, *n* (%)				0.75
No	4046(86.33)	3487(86.56)	559(86.05)	
Yes	808(13.45)	699(13.44)	109(13.95)	
Hypertension, *n* (%)				0.36
No	2974(64.56)	2575(64.77)	399(62.03)	
Yes	1897(35.44)	1626(35.23)	271(37.97)	
PIR, *n* (%)				< 0.001
<1	1137(16.26)	985(16.73)	152(24.55)	
>4	1120(33.14)	1007(36.57)	113(19.26)	
1–4	2225(44.53)	1882(46.70)	343(56.19)	

DM, diabetes mellitus; IFG, impaired fasting glucose; IGT, impaired glucose tolerance; NGT, normal glucose tolerance; PIR, poverty income ratio; CKD, chronic kidney disease; BMI, body mass index; TyG, triglyceride-glucose index; LTBI, latent tuberculosis infection

### Relationship between TyG and its related parameters and the occurrence of TB infection in different glucose metabolism groups

To further explore the correlation between TyG and its related parameters with the occurrence of TB infection across varying glucose metabolic statuses, we conducted multivariable logistic regression analysis in subgroups defined by their glucose metabolism (Table 3). After adjusting for covariates, our findings revealed individuals of different glucose metabolism statuses exhibited varying TB infection risk with TyG and its derivative parameters. In the population with NGT, levels of TyG (OR 2.17, 95%CI 1.40–3.35), TyG-WC (OR 1.01, 95%CI 1.00-1.01), and TyG-BMI (OR 1.02, 95%CI 1.00-1.04) were correlated with TB infection (all *P* < 0.05), while TyG-WHtR (OR 2.12, 95%CI 0.90–4.98, *P* = 0.08) was not associated with TB infection. In IFG participants, TyG (OR 57.10, 95%CI 1.17-278.66), TyG-WC (OR 1.02, 95%CI 1.00-1.05), TyG-WHtR (OR 872.94, 95%CI 43.31-17592.72) were significant with TB infection (all *P* < 0.05), while TyG-BMI (OR 1.10, 95%CI 0.91–1.32, *P* = 0.30) was not correlated. Conversely, in participants with IGT and DM, neither TyG index and its related parameters demonstrated a significant association with TB infection (*P* > 0.05).

**Table 3:** Multiple logistic analysis of TyG and related parameters and the occurrence of TB infection in different glucose metabolic status

TyG and related index	Glucose metabolic status	OR (95%CI)	*P* value	*P* for interaction
TyG				0.61
	DM	1.50(0.69,3.23)	0.28	
	IFG	57.10(1.17,2785.66)	**0.04**	
	IGT	2.91(0.41, 20.67)	0.27	
	NGT	2.17(1.40,3.35)	**0.002**	
TyG-WC				0.7
	DM	1.00(1.00,1.01)	0.48	
	IFG	1.02(1.00, 1.05)	**0.05**	
	IGT	0.99(0.98, 1.01)	0.49	
	NGT	1.01(1.00,1.01)	**0.01**	
TyG-BMI				0.49
	DM	1.01(0.98,1.03)	0.6	
	IFG	1.10(0.91, 1.32)	0.3	
	IGT	1.01(0.91, 1.12)	0.88	
	NGT	1.02(1.00,1.04)	**0.04**	
TyG-WHtR				0.8
	DM	2.00(0.69,5.86)	0.19	
	IFG	872.94(43.31,17592.72)	**< 0.001**	
	IGT	0.33(0.04, 2.81)	0.29	
	NGT	2.12(0.90,4.98)	0.08	

DM, diabetes mellitus; IFG, impaired fasting glucose; IGT, impaired glucose tolerance; NGT, normal glucose tolerance

Adjusted variables: age, HbA1c, fasting glucose, albumin, creatinine, triglycerides, cholesterol, races, educational levels, alcohol consumption, CKD, hypertension

### The RCS analysis of TyG and its related parameters and the occurrence of TB infection in different glucose metabolism groups

**Fig. 2:**
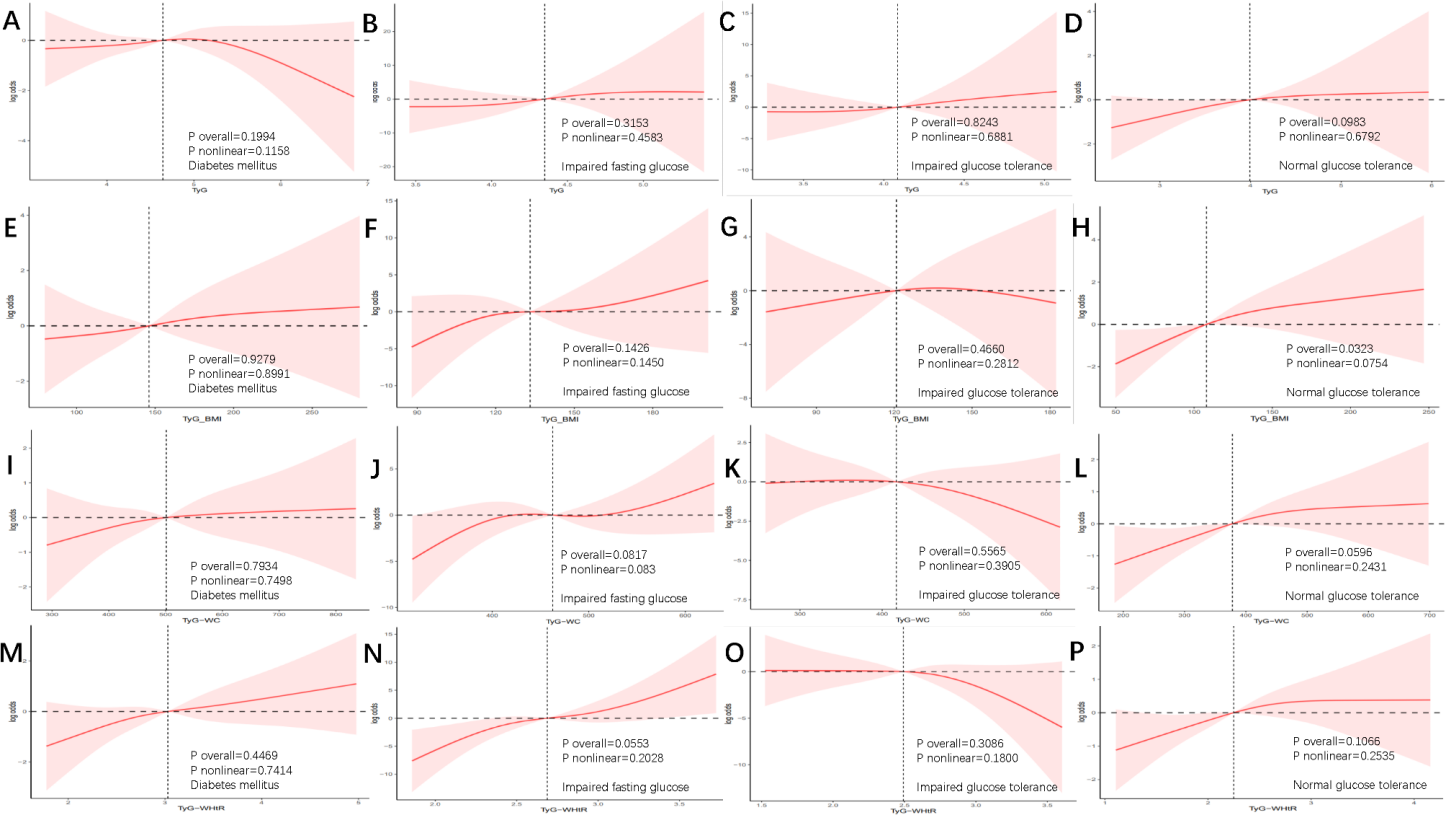
Restricted cubic spline plot of TyG and its derivative parameters with the occurrence of tuberculosis infection under different glucose metabolism statuses. Panels **A**–**D** represent the RCS curves of TyG index in individuals with different glucose metabolic statuses, respectively. Similarly, panels **E**–**H** represent TyG-BMI, panels **I**–**L** represent TyG-WC, panels **M–P** represent TyG-WHtR

To further elucidate the link between the TyG index and its derivative parameters with TB infection across different glucose metabolism statuses, we conducted an exploratory analysis using RCS and smooth curve-fitting logistic regression models, as shown in Fig. 2. After adjusting for confounding factors, including age, HbA1c, fasting glucose, albumin, creatinine, triglycerides, cholesterol, races, educational levels, alcohol consumption, CKD, hypertension, the relationship between the TyG index and its related parameters with the likelihood for TB infection was found to be approximately linear (all *p* values for non-linearity > 0.05).

### Relationship of TyG index with the likelihood of TB infection

Our analysis revealed that the TyG index was significantly elevated in the TB infection group (4.29 vs. 4.18, *P* = 0.01). To further investigate this association, we conducted a sensitivity analysis by converting the TyG index from a continuous to a categorical variable based on quartile distribution (Table 4). In the NGT group, the multivariable adjusted OR and 95%CI for TB infection across ascending TyG index quartiles were as follows: 1.00 (reference), 1.87 (1.24– 2.83), 1.89 (1.35–2.64), and 2.45 (1.31–4.60), respectively; In the IGT group, the adjusted OR for the TyG index quartiles were 1.00 (reference), 2.70 (0.90– 8.06), 29.42(4.72–183.19), and 761.33 (10.54–54999.02), respectively. No significant association between the TyG index and TB infection was observed in the DM and IFG groups in our study.

**Table 4:** Multiple logistic analysis of TyG and the occurrence of TB infection in different glucose metabolic status

TyG	Q1	Q2	Q3	Q4	*P* for trend (character2integer)	*P* for interaction
Glucose metabolic status						0.46
NGT	Reference	1.87(1.24,2.83) ***P*** **= 0.01**	1.89(1.35,2.64) ***P*** **< 0.001**	2.45(1.31,4.60) ***P*** **= 0.01**	**0.005**	
IGT	Reference	2.70(0.90, 8.06) *P* = 0.07	29.42(4.72, 183.19) ***P*** **= 0.001**	761.33(10.54,54999.02 ***P*** **= 0.005**	**0.001**	
DM	Reference	0.68(0.12,3.92) *P* = 0.65	0.79(0.18,3.49) *P* = 0.74	1.67(0.35,8.03) *P* = 0.5	0.18	
IFG	Reference	0.45(0.06, 3.50) *P* = 0.42	2.05(0.17,25.05) *P* = 0.55	4.05(0.20,82.64) *P* = 0.34	0.27	

DM, diabetes mellitus; IFG, impaired fasting glucose; IGT, impaired glucose tolerance; NGT, normal glucose tolerance

Adjusted variables: age, HbA1c, fasting glucose, albumin, creatinine, triglycerides, cholesterol, races, educational levels, alcohol consumption, CKD, hypertension

## Discussion

In this cross-sectional analysis of 4823 adults, several significant associations between the TyG index and TB infection were identified, which are as follows: (1) in individuals with NGT, we observed a correlation between TB infection and levels of TyG, TyG-WC and TyG-BMI; (2) In IFG participants, an association was found between TB infection and TyG, TyG-WC, TyG-WHtR; (3) a higher TyG index was independently linked to an increased likelihood of TB infection in individuals with NGT and IGT. These findings are of considerable interest. The TyG index, along with its related parameters, as a predictive tool for TB infection in individuals with NGT or in a prediabetic state. However, this predictive value does not extend to those with established DM.

TB infection and hyperglycemia share a complex and interconnected relationship. Firstly, due to stress, prolonged inflammation [16], glucose metabolic status and IR caused by TB infection [17], many patients without previous DM history have been observed to develop hyperglycemia at the onset of TB treatment [18]. Secondly, a hyperglycemic state can impair immune function, which may manifest as compromised neutrophil activity, a suppressed antioxidant system, and diminished humoral immunity [19]. Such immunological dysregulation can increase susceptibility to infection. Moreover, DM is known to elevate the risk of TB infection [6], the development of multi-drug-resistant tuberculosis [20], and is correlated with adverse treatment outcomes [21], higher rates of treatment failure, and delayed sputum conversion [22].

Aside from DM, evidence suggests that elevated fasting plasma glucose levels also contribute to the burden of TB infection [23], which is consistent with our study that participants with TB infection had higher levels of HbA1c, fasting glucose, along with a greater prevalence of disrupted glucose metabolism. Pre-DM, marked by an intermediate state of hyperglycemia and characterized by IR, is increasingly recognized globally, particularly in TB endemic regions [6]. The prevalence of pre-DM is estimated about 15–30% [24, 25]. A prospective cohort study in South Africa, enrolled non-DM pulmonary tuberculosis patients, revealed an IR prevalence of 25.4% [26]. IR is associated with low-grade systemic inflammation, stemming from factors such as adipokine deregulation and adipose tissue inflammation, which significantly influence in innate immunity [27]. This inflammation results in increased circulating levels of innate immune markers, including interferon-β, pro-inflammatory cytokines (TNF-α, IL-1β and IL-6) and anti-inflammatory cytokines (IL-10, IL-1Ra and TGF-β) [28]. TNF-α, IL-1β and IL-6 are known for their role in macrophages activation and mycobacterial growth restriction. However, an animal study noted that in the setting of IR, macrophage obtained a unique M2-like phenotype, enhancing glycolysis [29], which may dampen inflammatory responses and suppress the antibacterial actions necessary for pathogens clearance in polymicrobial sepsis. These complicated effects of IR on innate immunity can impact the elimination and reactivation of Mycobacteria tuberculosis (*M.tb*). In our study, the IR indicators-TyG index and associated parameters were associated with TB infection in NGT and Pre-DM individuals, aligning with previous study. An observational study enrolled 804 subjects revealed a higher prevalence of LTBI in NGT participants compared to those with pre-DM and newly diagnosed DM patients [30]. This disparity may be due to increased IL-1β and adiponectin levels and decreased IFN-γ secretion in DM patients. Conversely, we observed no correlation between the TyG index and associated parameters with TB infection in DM patients. Generally, IR can enhance trained immunity in macrophages through AKT signaling pathway, offering protection against TB [31]. However, these beneficial effects are diminished in chronic diabetes condition [29]. Therefore, during hyperglycemia status and chronic inflammatory states induced by DM, the immune cell responses and cytokine profiles are altered. Additionally, *M. tb* infected macrophages are known to constitutively secrete high levels of IL-10 [32], which can mitigate IR [28]. It is important to note that our study did not dynamically monitor blood glucose levels and the timing of diabetes diagnosis.

As a component of the TyG index, triglyceride may modulate immune cell function and polarization [33], potentially leading to dysregulated immune responses and contributing to TB risk. Higher baseline triglyceride levels have been associated with treatment failure in TB patients [34]. However, the role of triglyceride in TB pathogenesis is complex. For instance, an observational study reported that TB patients exhibited lower triglyceride levels alongside more severe inflammation [35], highlighting the multifaceted relationship between triglycerides and immune function. Additionally, as an indicator of nutritional status, triglycerides are linked to BMI. Underweight individuals, often presenting with low triglyceride levels, are at a higher risk of TB [36] and experience worse outcomes [37]. Our study found that TyG index and its derivative parameters were associated with TB risk in individuals with NGT and pre-DM, but not in those with established DM. This suggests that impaired glycemic control plays a more prominent role in TB susceptibility than triglyceride components. The stronger association of TB infection with glucose dysregulation indices, such as TyG-WC and TyG-WHtR, further supports this hypothesis. Both dysglycemia and hypertriglyceridemia are key components of metabolic syndrome [38], which is linked to an increased risk of cardiovascular diseases and type 2 DM [39]. The TyG index, as a marker of IR, reflects the interplay of these metabolic derangements. Given the association between IR, metabolic syndrome, and immune dysfunction, addressing these factors through lifestyle modifications and medical interventions may contribute to reducing TB incidence and transmission.

A key strength of our study lies in its foundation on the NHANES database, which provides a nationally representative sample of the U.S. population. This robust platform allowed us to explore the TyG index and its derived parameters in relation to TB infection across different glucose metabolic states. With a large size, our study representing 200 million non-institutionalized civilians, ensuring the reliability and generalizability of our results. Additionally, we further enhanced the reliability and applicability of our findings by employing appropriate NHANES sample weights during data analysis. Additionally, we meticulously adjusted covariates to address potential confounding biases, thereby bolstering the robustness of our results. The TyG index, requiring only triglycerides and fasting blood glucose along with easily obtained body measurement indicators, renders our study’s findings highly accessible for large-scale epidemiological studies. This is particularly significant given the increased risk of TB in the early stages of DM and pre-DM, where NGT is often overlooked. Our study provides significance for conducting early TB screening through easy clinical parameters. However, our study is not without limitations. As a retrospective analysis, we cannot establish causality between the variables examined. The single measurement of various indicators precludes insights into their continuous longitudinal changes and clinical outcome correlations. Future longitudinal studies or prospective cohort studies are needed to determine whether TB infection directly leads to changes in glucose metabolism or if pre-existing dysglycemia increase TB susceptibility. Furthermore, the study did not differentiate in detail between type 1 and type 2 diabetes, which may introduce some bias into the final results. The generalizability of our findings may be limited as the study exclusively included the American population. Given the known differences in ethnicity, diet, exercise, and sleep habits, further validation of our results in diverse cohorts from multiple regions with varying TB prevalence and metabolic risk profiles are warranted. Lastly, while we adjusted for numerous covariates, there may be potential confounders that were not fully accounted for in this study, and these covariates may influence clinical outcomes in a dynamic manner rather than remaining constant. Further large-scale, multicenter studies employing longitudinal designs or dynamic modeling approach are warranted to validate the findings of this study and to investigate additional factors influencing the relationship between glucose metabolism, the TyG index, and TB infection.

## Conclusions

Our study found that in the population with NGT and IFG, levels of TyG index and associated parameters were associated with TB infection. Higher TyG index was independently associated with an increased likelihood of TB infection in NGT and IGT individuals, while not in DM and IFG participants.

## Data Availability

Publicly available datasets were analyzed in this study. These data can be found here: https://www.cdc.gov/nchs/nhanes/index.htm.

## Abbreviations

BMI: Body mass index
CKD: Chronic kidney disease
DM: Diabetes mellitus
HbA1c: Glycated hemoglobin
HOMA-IR: Homeostasis model assessment of insulin resistance
IFN-γ: Interferon-gamma
IFG: Impaired fasting glucose
IGRA: Interferon-Gamma Release Assays
IGT: Impaired glucose tolerance
IR: Insulin resistance
LTBI: Latent tuberculosis infection
*M.tb*: Mycobacteria tuberculosis
NHANES: National Health and Nutrition Examination Survey
NGT: Normal glucose tolerance
OGTT: Oral glucose tolerance test
PIR: Poverty income ratio
PPD: Purified Protein Derivative
QFT-GIT: QuantiFERON-TB Gold In-Tube
RCS: Restricted cubic splines
TB: Tuberculosis
TyG: Triglyceride-glucose
TyG–WC: Triglyceride-glucose-waist circumference
TyG-BMI: Triglyceride-glucose–body mass index
TyG-WHtR: Triglyceride-glucose-waist-to-height ratio
